# Deoxyuridine triphosphatase (dUTPase) expression and sensitivity to the thymidylate synthase (TS) inhibitorD9331

**DOI:** 10.1054/bjoc.2000.1358

**Published:** 2000-08-17

**Authors:** S D Webley, A Hardcastle, R D Ladner, A L Jackman, G W Aherne

**Affiliations:** CRC Centre for Cancer Therapeutics at the Institute of Cancer Research, 15 Cotswold Road, Sutton, Surrey, SM2 5NG, UK; Department of Molecular Biology, University of Medicine and Dentistry of New Jersey, School of Osteopathic Medicine, 2 Medical Center Drive, Stratford, NJ, 08084, USA

## Abstract

Uracil DNA misincorporation and misrepair of DNA have been recognized as important events accompanying thymidylate synthase (TS) inhibition. dUTPase catalyses the hydrolysis of dUTP to dUMP, thereby maintaining low intracellular dUTP. We have addressed the relationship between dUTPase expression and cellular sensitivity to TS inhibition in four human lung tumour cell lines. Sensitivity (5-day MTT assay) to the growth inhibitory effects of the non-polyglutamatable, specific quinazoline TS inhibitor ZD9331, varied up to 20-fold (IC _50_ 3–70 nM). TS protein expression correlated with TS activity (*r*^2^= 0.88 *P*= 0.05). Intracellular concentrations of drug following exposure to ZD9331 (1 μM, 24 h) varied by ~2-fold and dTTP pools decreased by > 80% in all cell lines. No clear associations across the cell lines between intracellular drug concentrations, TS activity/expression, or TTP depletion could be made. dUTPase activity varied 17-fold and correlated with dUTPase protein expression (*r*^2^= 0.94 *P*= 0.03). There was a striking variation in the amount of dUTP formed following exposure to ZD9331 (between 1.3 and 57 pmole 10^–6^cells) and was in general inversely associated with dUTPase activity. A large expansion in the dUTP pool was associated with increased sensitivity to a 24-h exposure to ZD9331 in A549 cells that have low dUTPase activity/expression. dUTPase expression and activity were elevated (approximately 3-fold) in two variants of a human lymphoblastoid cell line with acquired resistance to TS inhibitors, further suggesting an important role for this enzyme in TS inhibited cells. © 2000 Cancer Research Campaign


*British Journal of Cancer* (2000) **83**(6), 792–799
© 2000 Cancer Research Campaign
doi: 10.1054/ bjoc.2000.1358, available online at http://www.idealibrary.com on IDEAL®

### Deoxyuridine triphosphatase (dUTPase) expression and sensitivity to the thymidylate synthase (TS) inhibitor ZD9331

**SD Webley[1], A Hardcastle[1], RD Ladner[2], AL Jackman[1] and GW Aherne[1]**

[1]CRC Centre for Cancer Therapeutics at the Institute of Cancer Research, 15 Cotswold Road, Sutton, Surrey, SM2 5NG, UK; [2]Department of Molecular Biology, University of Medicine and Dentistry of New Jersey, School of Osteopathic Medicine, 2 Medical Center Drive, Stratford, NJ 08084, USA.

**Summary** Uracil DNA misincorporation and misrepair of DNA have been recognized as important events accompanying thymidylate synthase (TS) inhibition. dUTPase catalyses the hydrolysis of dUTP to dUMP, thereby maintaining low intracellular dUTP. We have addressed the relationship between dUTPase expression and cellular sensitivity to TS inhibition in four human lung tumour cell lines. Sensitivity (5-day MTT assay) to the growth inhibitory effects of the non-polyglutamatable, specific quinazoline TS inhibitor ZD9331, varied up to 20-fold ($IC_{50}$ 3–70 nM). TS protein expression correlated with TS activity ($r^2 = 0.88$, $P = 0.05$). Intracellular concentrations of drug following exposure to ZD9331 (1 µM, 24 h) varied by ~2-fold and dTTP pools decreased by > 80% in all cell lines. No clear associations across the cell lines between intracellular drug concentrations, TS activity/expression, or TTP depletion could be made. dUTPase activity varied 17-fold and correlated with dUTPase protein expression ($r^2 = 0.94$, $P = 0.03$). There was a striking variation in the amount of dUTP formed following exposure to ZD9331 (between 1.3 and 57 pmole $10^{-6}$ cells) and was in general inversely associated with dUTPase activity. A large expansion in the dUTP pool was associated with increased sensitivity to a 24-h exposure to ZD9331 in A549 cells that have low dUTPase activity/expression. dUTPase expression and activity were elevated (approximately 3-fold) in two variants of a human lymphoblastoid cell line with acquired resistance to TS inhibitors, further suggesting an important role for this enzyme in TS inhibited cells. © 2000 Cancer Research Campaign

**Keywords**: dUTPase; thymidylate synthase; ZD9331; dUTP; in vitro sensitivity

Thymidylate synthase (TS) represents a key enzyme in pyrimidine biosynthesis catalyzing the reductive methylation of deoxyuridylate (dUMP) to thymidylate (dTMP). The transferred methyl group is donated by 5,10-methylene tetrahydrofolate ($N^5$,$N^{10}$-$CH_2FH_4$). This reaction provides the sole intracellular de novo source of dTMP that is used exclusively in the biosynthesis and repair of DNA. This unique function of TS has led to the development of novel folate-based anti-tumour compounds that target it specifically (Jackman and Judson, 1996).

The activity of these and other clinically used TS inhibitors have been shown to be influenced by a number of factors, including drug uptake and activation (Jackman et al, 1995) and inactivation (Rhee et al, 1993). Amplification of the TS gene is an important determinant of acquired resistance to TS inhibitors in vitro (Jackman et al, 1986, O'Connor et al, 1992; Copur et al, 1995; Freemantle et al, 1995; Jackman et al, 1995). Modulation of TS expression by using antisense TS RNA has recently been reported to influence cell sensitivity to fluoropyrimidines and antifolates (Kobayashi et al, 1995; Ju et al, 1998). In the clinic, a correlation between both TS protein and TS gene transcript levels of colorectal, gastric and breast carcinoma and response to 5-FU-based chemotherapy for advanced disease (Johnston et al, 1994; 1995; 1997; Lenz et al, 1995; Pestalozzi et al, 1997) has been reported. However, no link was found between TS expression in primary tumour and response to TS-directed chemotherapy for subsequent metastatic disease (Findlay et al, 1997). This could be explained by different levels of TS expression in metastatic lesions compared to primary tumours. However, the expression of other proteins involved in drug uptake and metabolism, or in downstream events such as DNA repair and cell death mechanisms, may also have a role to play in determining sensitivity to TS inhibition.

Inhibition of TS leads to rapid depletion in the dTTP pool and an expansion in the dUMP pool (Jackson et al, 1983; Aherne et al, 1996*a*). This in turn may be phosphorylated to dUTP. These dNTP perturbations are thought to be important in the eventual loss of cell viability following TS inhibition. As DNA polymerase does not distinguish between dUTP and dTTP, the accumulation in dUTP during dTTP depletion promotes the misincorporation of the fraudulent base, uracil, into DNA where it is subsequently excised by uracil-DNA-glycosylase to form an apyrimidinic site. If dTTP remains depleted, dUTP may be reinserted and a futile cycle of excision repair and re-insertion will occur leading to lethal DNA damage. This 'misincorporation theory' (reviewed in Aherne and Brown, 1999) has provided a possible mechanism of action promoting cell death in dTTP-depleted cells (Curtin et al, 1991; Canman et al, 1993).

dUTPase is an ubiquitous enzyme that is responsible for the hydrolysis of dUTP to dUMP thereby minimizing the misincorporation of dUTP into DNA (Lindahl, 1979). Recent studies using tumour cells with genetically increased dUTPase activity that exhibit enhanced resistance to FdUrd have further supported the belief that dUTPase, the main regulator of dUTP pools, serves an

*Received 6 January 2000*
*Revised 16 May 2000*
*Accepted 17 May 2000*

*Correspondence to*: GW Aherne important role in mediating the effects of TS inhibitors (Canman et al, 1994). Thus, we have studied the expression of this enzyme in different human tumour cell lines and addressed the relationship between dUTPase expression and cellular response to TS inhibition.

Historically, the fluorinated pyrimidines have been used extensively to study the critical events leading from TS inhibition to cell death (Aherne and Brown, 1999). However, since metabolites of these compounds may be incorporated into nucleic acids the cellular effects of thymineless death cannot necessarily be attributable solely to a thymineless state. ZD9331 is a quinazoline-based antifolate-specific TS inhibitor (Jackman et al, 1997) currently under clinical development. Since ZD9331 acts only at the TS loci, this compound has provided an appropriate tool to study the cellular effects of TS inhibition. In addition, the lack of folylpolyglutamylsynthetase (FPGS) substrate activity for ZD9331 prevents its cellular retention (due to a lack of polyglutamate formation), hence use of ZD9331 also permits greater control of the duration of TS inhibition (Aherne et al, 1996*b*).

### MATERIAL AND METHODS

### Materials

All standard laboratory chemicals used in this study were of AnalaR grade and purchased either from British Drug Houses (BDH, Poole, Dorset, UK) or from Sigma (Poole, Dorset, UK). ZD9331, raltitrexed (RTX, Tomudex, ZD1694), nolatrexed (Thymitaq, AG337 (Webber et al, 1996)) and CB300179 (Skelton et al, 1998) were synthesized at Zeneca Pharmaceuticals (Macclesfield, Cheshire, UK). ‘Tomudex’ is a trademark. Property of Zeneca Limited, part of AstraZeneca. ZD9331 and RTX were prepared in 0.15 M sodium bicarbonate ($NaHCO_3$), AG337 and CB300179 were dissolved in 100% dimethysulphoxide (DMSO) to give a 20 mM solution and solutions were filter sterilized. Deoxythymidine 5′–triphosphate, [methyl-$^3$H] tetra sodium salt (NET-221X-specific activity 78 Ci mmole$^{-1}$) was purchased from New England Nuclear (NEN) Research Products. [5-$^3$H]-deoxyuridine 5′-monophosphate, ammonium salt (TRK287-specific activity 15.4 Ci mmole$^{-1}$) and [$^3$H] deoxyuridine 5′-triphosphate (TRK 351-specific activity 22 Ci mmol$^{-1}$) were obtained from Amersham International plc (Little Chalfont, Bucks, UK).

### Cell culture

The human lung carcinoma cell lines A549 (squamous), CORL23 (large cell), MOR (adenocarcinoma) and HX147 (large cell) were maintained in DMEM tissue culture medium (Gibco, Paisley, Scotland) and 10% heat inactivated dialysed fetal bovine serum (FBS) (Imperial Laboratories, Andover, Hants, UK) supplemented with 2 mM glutamine, 50 μg ml$^{-1}$ gentamycin, 2.5 μg ml$^{-1}$ fungizone, 10 μg ml$^{-1}$ insulin and 0.5 μg ml$^{-1}$ hydrocortisone, at 37°C in air containing 5% $CO_2$. The doubling times of these cell lines were 18, 28, 23, and 25 h respectively. All cells were routinely subcultured once a week. The cells were counted using a haemocytometer (Neubauer Weber, UK). The W1L2 human lymphoblastoid cell line was grown in suspension in RPMI 1640 containing 20 mM HEPES without $NaHCO_3$ and supplemented with 10% heat inactivated (56°C, 30 min) dialysed FBS (Imperial Laboratories or ICN Flow, Thames Oxfordshire UK), 20 μg ml$^{-1}$ gentamycin, 2 mM L-glutamine and 0.5 μg ml$^{-1}$ fungizone. Cells were maintained at 37°C. W1L2-resistant sub-lines (W1L2R$^{1694}$ (Jackman et al, 1995), and W1L2:C1 (O’Connor et al, 1992) that overexpress TS by 500- and 200-fold respectively, and W1L2R$^{179}$ (Kobayashi et al, 1995) and W1L2R$^{9331}$ (unpublished results) both with a ~20-fold increase in TS activity) were maintained in medium containing the selective compound (5 μM RTX, 50 μM ICI198583, 5 μM CB300179, or 0.02 μM ZD9331) until 2–3 weeks prior to experimentation, when they were grown in drug-free medium (DFM) in the same way as the parent line. Cell numbers were determined using a Coulter Counter, model ZM. All cell lines were free of mycoplasma during the course of these studies.

### Growth inhibition studies

The human lung tumour cell lines were seeded at 1–2 × $10^3$ cells per well in a 96-well tissue culture plate (Falcon) and left to adhere overnight prior to drug exposure. Cells were exposed to drugs for 120 h before cell growth inhibition analysis using the MTT assay. For 24 h $IC_{50}$ experiments, drug was removed 24 h after drug exposure using three PBS washes (37°C). The cells were incubated for a further 120 h in 200 μl culture medium before analysis using MTT. 50 μl MTT (5 mg ml$^{-1}$) (Sigma) was added to each well, the plates incubated at 37°C for 4 h and the medium aspirated. The resulting formazan crystals were dissolved using 100 μl of DMSO (BDH), the plates shaken for 10 min on a plate shaker and the absorbance measured at 540 nm on a Multiscan plate reader (Labsystems).

### Intracellular dTTP, dUMP and dUTP

The human lung tumour cell lines were seeded in T-80 tissue culture flasks (Falcon) in triplicate (5 × $10^5$ cells 10 ml$^{-1}$) and left to reach 50–60% confluency before treatment. Cells were exposed for 4, 16 or 24 h to medium containing ZD9331. After drug exposure, the medium was aspirated and flasks flooded with 1 ml ice-cold 0.4 M perchloric acid (PCA), the cells vortexed vigorously and left on ice for 30 min with intermittent vortexing before centrifugation (4°C, 20 min) at 3000 rpm (1500 ***g***). The supernatants were transferred to chilled Eppendorf tubes and neutralized with a half volume of 0.73 M KOH in 0.16 M $KHCO_3$. The neutralized samples were left on ice for 30 min then centrifuged at 10 000 rpm (2500 ***g***) for 10 min (4°C) and the resulting supernatant was stored at –70°C until further treatment.

Thawed cell extracts were treated with sodium periodate to remove NTPs and intracellular dUTP, dTTP and immunoreactive ‘dUMP’ measured in duplicate as previously described (Aherne et al, 1996*a*). Cell numbers were determined from parallel flasks and cellular dUTP, dTTP and ‘dUMP’ were expressed as pmole per $10^6$ cells.

### Measurement of intracellular ZD9331

Duplicate flasks of human lung cancer cells in logarithmic growth (50–60% confluency) were exposed to ZD9331 for 4 or 24 h and the cells washed twice with cold PBS. Cells were harvested by scraping into 1 ml of ice cold PBS before centrifuging for 5 min at 2500 rpm (600 ***g***) (4°C). The cell pellet was re-suspended in 1 ml

© 2000 Cancer Research Campaign 

of PBSGT, freeze-thawed twice, sonicated for 30 s (12 microns) (Soniprep 150 MSE), then centrifuged for 5 min (4°C, 2500 rpm). The supernatant was removed and stored at –20°C. Intracellular ZD9331 concentrations were determined using an antibody coated ELISA (Jackman et al, 1997).

### Western immunoblot analysis

Western blot analysis was performed according to standard protocols and the protein bands of interest were probed using either a rabbit polyclonal antibody (affinity purified) to recombinant human dUTPase (Ladner et al, 1996) or an affinity purified antiserum to human recombinant TS (Aherne et al, 1997). Recombinant proteins were used as controls (data not shown). Visualization of the protein bands was performed using enhanced chemiluminescence (ECL) reagents (Amersham International).

### TS assay

The method for the measurement of TS activity has been described previously (Calvert et al, 1980). The assay is based on the release of $^3H$ in the form of $H_2O$ from 5-[$^3H$]dUMP by TS. Small Dowex columns (anion exchanger) were utilized to separate the product ([$^3H$] $H_2O$) from the substrate (5-[$^3H$]dUMP). Briefly, cells in log phase (~$1 \times 10^7$) were resuspended in 2 ml of TS buffer (0.125 mM $KH_2PO_4$, 3 mM dithiothreitol, pH 7.4). The cell suspension was then sonicated at 12 microns for three separate 10 s intervals on ice then centrifuged for 1 h at 19 000 rpm (50 000 ***g***) at 4°C. TS activity was measured in the resulting supernatant in a 0.5 ml incubation mixture containing: 0.2 ml of assay mix (containing 10 µl [$^3H$]dUMP (~0.5 µCi), 2.5 µl 10 mM 'cold' dUMP, and 187.5 µl $H_2O$), and 0.05 ml 2 mM L-$N^5$ $N^{10}$-$CH_2FH_4$. The reaction was allowed to proceed for 1 h at 37°C and terminated by the addition of 1 ml of iced water. The sample was than passed down a 3 × 0.5 cm Dowex column and eluted with a further 2 ml of iced water. The amount of radioactivity in the effluent was counted using scintillation counting. Protein estimations were performed using the Pierce BCA kit according to manufacturer's instructions. TS activity was calculated from three supernatant dilutions (linear up to the highest concentration used) with and without $FH_4$, and expressed as nmole product per mg protein per h.

### dUTPase assay

The dUTPase assay (Caradonna and Adamkiewicz, 1984) is based on the formation of [$^3H$]dUMP from [$^3H$]dUTP. Separation of radiolabeled dUMP, dUDP and dUTP after the reaction was achieved by thin layer chromatography (TLC). Briefly, exponentially growing cells (~$1 \times 10^6$) were resuspended in 0.5 ml dUTPase extraction buffer containing 50 mM Tris (pH 7.6 at 37°C) and 2 mM DTT. The cells were frozen to –80°C for 10 min, thawed, sonicated twice for 30 s (10 microns) then centrifuged for 10 min at 18 000 rpm (9000 ***g***) (4°C). The resulting supernatant was then assayed for dUTPase. Protein estimations were performed using the Pierce BCA kit according to manufacturer's instructions. 1 unit of dUTPase activity was expressed as nmole dUTP hydrolysed $min^{-1}$ $mg^{-1}$ of protein at 37°C.

### Statistical analysis

All statistical analyses were performed using GraphPad Prism software. The Spearmans non-parametric correlation test was used to determine the linear association between the rank order of two variables. The square of the correlation coefficient ($r^2$) calculated from this test quantified the direction and magnitude of the correlation. The paired Students t-test and one-way ANOVA were used to investigate differences between variables.

### RESULTS

### Growth inhibitory activity of ZD9331

The effect on cell growth over a 120 h period of continuous exposure to ZD9331 in the human lung carcinoma cell line panel is shown in Table 1. The concentration of ZD9331 required for inhibition of growth by 50% ($IC_{50}$) varied up to 20-fold. The least sensitive cell line was A549 ($IC_{50}$ = 70 ± 14.7 nM) which compared to an $IC_{50}$ of 3.2 (1 nM for the most sensitive cell line, CORL23). A similar pattern of sensitivity was obtained with the highly polyglutamatable TS inhibitor, RTX, and with the lipophilic TS inhibitor, AG337 (Table 1). A549 cells were ~8- and ~20-fold less sensitive to RTX and AG337 respectively than CORL23 and MOR cells.

All cell lines were more sensitive to RTX than ZD9331 (ZD9331 $IC_{50}$: RTX $IC_{50}$ ratio was 2:9). Since RTX and ZD9331 both use the RFC for cell entry and both target TS, the higher relative sensitivity of A549 and MOR cells to ZD1694 could indicate higher FPGS activity compared to CORL23 and HX147 cells. AG337 is at least 100-fold less active compared to ZD9331, an observation which can be accounted for by its lack of active cellular uptake and lower TS potency (Webber et al, 1996).

The pattern of sensitivity following a 24 h exposure to ZD9331 was different to that following a 120 h exposure (Table 1) and there was only a 3-fold variation in $IC_{50}$ concentrations. MOR cells which were 10-fold more sensitive than A549 cells after a 120 h exposure, were 2.5-fold less sensitive following a 24 h exposure.

**Table 1** ZD9331, ZD1694 and AG337 $IC_{50}$ values (mean ± SD) in four human lung tumour cell lines

| Cell line | ZD9331 120 h $IC_{50}$ nM | RTX 120 h $IC_{50}$ nM | ZD9331 $IC_{50}$:RTX $IC_{50}$ ratio | ZD9331 24 h $IC_{50}$ nM | ZD9331 24 h:120 h $IC_{50}$ ratio | AG337 120 h $IC_{50}$ µM |
|---|---|---|---|---|---|---|
| A549 | 70 ± 14.7 | 8.1 ± 4.6 | 8.6 | 111 ± 25 | 1.6 | 15.5 ± 5 |
| HX147 | 14 ± 5 | 4 ± 1.2 | 3.5 | 173 ± 75 | 12.3 | 5.53 ± 0.64 |
| CORL23 | 3.2 ± 1 | 1.4 ± 0.41 | 2.3 | n/e | n/e | 0.51 ± 0.3 |
| MOR | 7.3 ± 1.7 | 1 ± 0.45 | 6.7 | 276 ± 25 | 38 | 0.71 ± 0.2 |

Growth inhibition was determined using MTT assay (three separate determinations in quadruplicate); n/e = not evaluable

 © 2000 Cancer Research Campaign

**Table 2** Intracellular ZD9331 levels following exposure to 1 μM ZD9331 measured by ELISA

| Cell line | ZD9331 pmole $10^{-6}$ cells (4 h) | ZD9331 pmole $10^{-6}$ cells (24 h) |
|---|---|---|
| A549 | 16.5 ± 11 | 13.3 ± 3.9 |
| HX147 | 14.4 ± 6.8 | 11.6 ± 6.0 |
| CORL23 | 10.6:6.9 | 8.7:7.1 |
| MOR | 7.4 ± 1.1 | 6.3 ± 1.3 |

Mean ± SD of three separate experiments in duplicate except CORL23 where *n* = 2 in duplicate

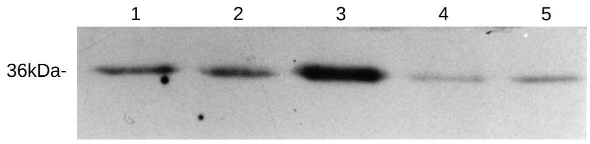

**Figure 1** TS protein expression in four human lung tumour cell lines determined by Western blotting. Lane 1 Recombinant TS; Lane 2 CORL23; Lane 3 HX147; Lane 4 MOR; Lane 5 A549. Expression was quantified using densitometry

**Table 3** Activity of TS and dUTPase in four human tumour cell lines

| Cell line | TS activity nmole product $h^{-1}$ $mg^{-1}$ protein | dUTPase activity nmole dUTP hydrolysed $min^{-1}$ mg $protein^{-1}$ |
|---|---|---|
| A549 | 2.9 ± 0.9 | 5.0 ± 2.0 |
| HX147 | 6.3 ± 0.5 | 1.2 ± 0.15 |
| CORL23 | 0.67:1.1 | 9.0:6.0 |
| MOR | 0.43 ± 0.12 | 17.2 ± 4.8* |

Values are mean ± SD of three experiments in triplicate (except CORL23 where *n* = 2); *dUTPase activity in MOR cells was significantly ($P$ < 0.001) higher than HX147 and A549 cells

The 24 h $IC^{50}$:120 h $IC_{50}$ ratio is shown in Table 1. The high ratio for MOR cells (38) was in contrast to that for A549 cells (1.6) which appeared to be more equally affected by a short and prolonged exposure to the drug.

### Intracellular concentrations of ZD9331

To establish that variations in sensitivity to ZD9331 were not due to differences in drug accumulation, intracellular drug levels were measured following a 4 h and a 24 h exposure to 1 μM ZD9331 (Table 2). ZD9331 concentrations at 4 h varied ~2-fold (7.4 ± 1.1 to 16.5 ± 11 pmole $10^{-6}$ cells) and were not significantly different. At 24 h drug levels were similar to those obtained after a 4 h exposure to ZD9331. A549 cells had the highest drug concentrations (13.3 ± 3.9 pmole $10^{-6}$ cells) and MOR cells the lowest (6.3 ± 1.3 pmole $10^{-6}$ cells) but this difference was not significant ($P > 0.05$). As may have been expected there was no relationship between intracellular levels of ZD9331 and sensitivity to either short (24 h) or prolonged (120 h) exposure to ZD9331.

### TS protein expression and activity

There was a 26-fold variation in TS protein expression (Figure 1) in the lung cell line panel as determined by Western blotting. The relative volume of integration obtained by densitometry ($n$ = 2) was 13, 338, 45, and 60 for CORL23, HX147, MOR, and A549 cells, respectively. TS protein expression and TS activity (Table 3) correlated well ($r^2 = 0.88$, $P = 0.05$). HX147 cells had significantly ($P < 0.001$) higher TS activity than MOR and CORL23 cells.

As the number of cell lines was small it was not possible to correlate TS activity with sensitivity to ZD9331 at 24 h or 120 h. However, it can be observed that although the two most sensitive cell lines following 120 h exposure to drug (CORL23 and MOR) had low TS activity, HX147 cells with the highest TS activity were 5-fold more sensitive to ZD9331 than A549 cells. However, HX147 cells accumulated more ZD9331 in 24 h than A549 cells.

### Effect of ZD9331 on dTTP, dUMP and dUTP pools

All cells showed a significant (at least $P < 0.05$) reduction (> 80%) in dTTP pools by 4 h (Table 4) confirming TS inhibition following 1 μM ZD9331. Interestingly, at 4 h dTTP depletion in HX147 cells (43% control) was significantly less ($P < 0.001$) than in A549 (13% control) and MOR (11% controls) cells and in CORL23 cells (21%, $P$ = <0.01). This may reflect the finding that HX147 had high TS activity and protein expression. Conversely, dTTP depletion was greatest in the cell line (MOR) with the lowest TS activity. Following 16 h and 24 h in drug, dTTP pools were depleted further in all cell lines except A549. A significant ($P < 0.0001$) rise in 'dUMP' pools was measured in all cells.

In contrast, large differences between cell lines in dUTP accumulation were observed (Figure 2). By 4 h, A549 cells had accumulated a significant ($P < 0.0001$) amount of dUTP (6.2 ± 0.9 pmole $10^{-6}$ cells) (control value in all cell lines was ~1 pmole $10^{-6}$ cells). By 24 h, the pool size increased to 57 ± 20 pmole dUTP $10^{-6}$ cells. The accumulation of dUTP in HX147 cells was slower than that in A549 cells, but by 24 h the pool had increased to 17 ± 8.6 pmole dUTP $10^{-6}$ cells. In contrast, MOR and CORL23 cells did not accumulate dUTP after ZD9331 treatment. Indeed, even at a 10-fold higher dose of ZD9331 (10 μM, 24 h) dUTP pools were only moderately increased (4.3 pmoles $10^{-6}$ cells) in MOR cells. This is in contrast to A549 cells in which dUTP pools were increased to 123 pmoles $10^{-6}$ cells under the same conditions (data not shown).

If the extent of dUTP accumulation were an important determinant of cellular response to ZD9331, then one would expect to observe a negative association between dUTP accumulation and $IC_{50}$. Although only three cell lines were included, this expected association was observed with 24 h $IC_{50}$ values (Figure 3) but not the 120 h continuous exposure sensitivity to ZD9331.

### dUTPase protein expression and activity

A 17-fold variation in dUTPase protein expression was observed in the lung tumour cell line panel (Figure 4A). dUTPase activity (Table 3) correlated ($P = 0.03$, $r^2 = 0.94$) with dUTPase protein expression (quantified using densitometry) (Figure 4B). MOR cells had significantly ($P < 0.001$) higher levels of dUTPase activity than HX147 and A549 cells. No association was observed between sensitivity (24 h or 120 h $IC_{50}$) to ZD9331 and dUTPase activity. Although a statistically significant correlation was not observed between dUTPase activity and the amount of dUTP

*© 2000 Cancer Research Campaign* 

**Table 4** dTTP and 'dUMP' pools following 1 µM ZD9331 in four human lung tumour cell lines

| | dTTP (pmole $10^{-6}$ cells) | | | | dUMP (pmole $10^{-6}$ cells) | | | |
|---|---|---|---|---|---|---|---|---|
| **Cell line** | **0 h** | **4 h** | **16 h** | **24 h** | **0 h** | **4 h** | **16 h** | **24 h** |
| A549 | 35.0 ± 7.9 | 4.5 ± 0.43 | 4.6 ± 1.2 | 5.7 ± 2.6 | 63.5 ± 16 | 184 ± 12 | 1673 ± 622 | 2899 ± 529 |
| HX147 | 31.6 ± 8.7 | 13.5 ± 1.8 | 8.5 ± 2.9 | 3.1 ± 1.2 | 58.0 ± 14 | 187 ± 14 | 201 ± 61 | 1331 ± 377 |
| CORL23 | 30.0 ± 2.4 | 6.3 ± 2.4 | 1.0 ± 03 | 1.6 ± 0.5 | 66.0 ± 3.4 | 361 ± 135 | 2648 ± 550 | 1848 ± 687 |
| MOR | 29.3 ± 3.1 | 3.15 ± 1.1 | 2.2 ± 1.8 | 1.7 ± 0.7 | 35.7 ± 9.3 | 135 ± 3.1 | 394 ± 113 | 1881 ± 383 |

Mean ± SD values are shown (three experiments in duplicate)

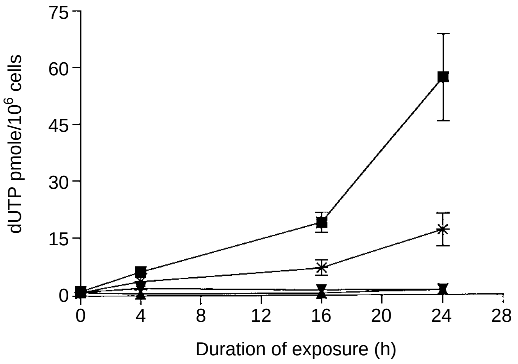

**Figure 2** Accumulation of dUTP following 1 µM ZD9331 in four human lung tumour cell lines: A549 (■), HX147 (*), MOR (▲) and CORL23 (▼). Mean ± SD of three experiments carried out in duplicate. A549 and HX147 accumulated significant ($P < 0.0001$ and $P < 0.05$ respectively) levels of dUTP by 4 h and 16 h respectively

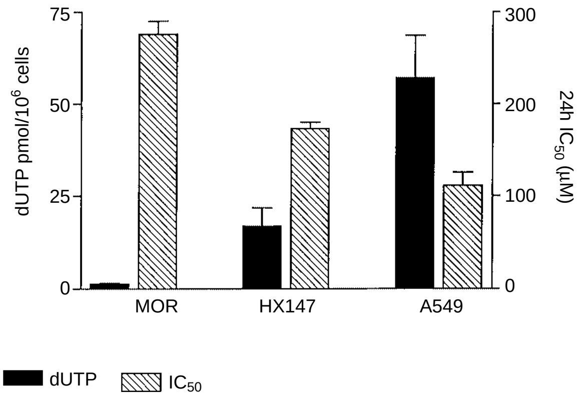

**Figure 3** Relationship between $IC_{50}$ (24 h) and dUTP accumulation formed, the two cell lines that did not accumulate dUTP (MOR and CORL23) had the highest dUTPase activity. dUTPase protein expression showed a similar wide variation in a panel of six human colon tumour cell lines (data not shown).

### dUTPase expression in W1L2-resistant cell lines

The dUTPase expression in four W1L2 cell lines with acquired resistance to different TS inhibitors was also studied. A 5.6- and 2-fold increase in dUTPase protein expression was observed in W1L2R$^{1694}$ and W1L2R$^{300179}$-resistant cell lines respectively (Figure 5A). Increased expression of dUTPase protein in these lines was associated with an increase in dUTPase activity. A significant ($P < 0.01$ and $P < 0.05$) increase (2.3-fold and 2.7-fold compared to the parent cell line) in dUTPase activity was measured in W1L2R$^{1694}$ and W1L2R$^{300179}$, respectively (Figure 5B). Protein expression in W1L2:C1 was similar to that of W1L2 itself. W1L2R$^{9331}$, which also showed no increase in dUTPase protein by Western blotting, had a similar level of dUTPase activity as the parent line.

### DISCUSSION

The aim of this study was to investigate the role of dUTPase expression in cellular response to TS inhibition. Four human lung carcinoma cell lines have been characterized with respect to sensitivity to the non-polyglutamatable TS inhibitor ZD9331, TS expression and activity, dUTPase expression and activity, and dNTP pool perturbations following ZD9331. The relative sensitivity of the cell lines to other TS inhibitors such as RTX and AG337 that have different biochemical profiles to ZD9331 has also been determined.

The 20-fold variation in sensitivity to ZD9331 (120 h exposure) in the four human lung tumour cell lines was not associated with differences in drug accumulation. Also, the ranking of cell sensitivity to AG337, which does not rely on the RFC for cell entry, was the same as for ZD9331. The pattern of sensitivity to RTX also mirrored that seen with ZD9331, except that the difference in the continuous exposure $IC_{50}$ value between the least sensitive (A549) and most sensitive (CORL23) cell line was not as great. A549 and MOR cells were relatively less sensitive to ZD9331 than RTX compared to CORL23 and HX147 cells. Response to AG337 may more closely reflect the effects of TS inhibition than response to RTX as AG337 has no requirement for the RFC or FPGS (Webber et al, 1996). It appears likely that a higher FPGS activity may account for the high relative sensitivity of A549 and MOR cells to RTX.

The 120 h continuous exposure $IC_{50}$ value for ZD9331 in A549 cells was similar to its 24 h $IC_{50}$ value (ratio = 1.6). This was in contrast to HX147 and MOR cells with 24 h $IC_{50}$:120 h $IC_{50}$ ratios of 37 and 12.3 respectively. This would suggest that there is a critical event(s) that causes significant growth inhibition in the first 24 h of ZD9331 treatment in A549 cells, whereas with MOR and HX147 cells a longer duration of drug exposure and TS inhibition appears to be necessary before this lesion(s) occurs.

Alternatively, A549 cells may be less sensitive to events that occur later during prolonged periods of dTTP depletion following exposure to ZD9331. Thus, the duration of TS inhibition required to cause a significant growth inhibitory response is cell line-dependent

 © 2000 Cancer Research Campaign

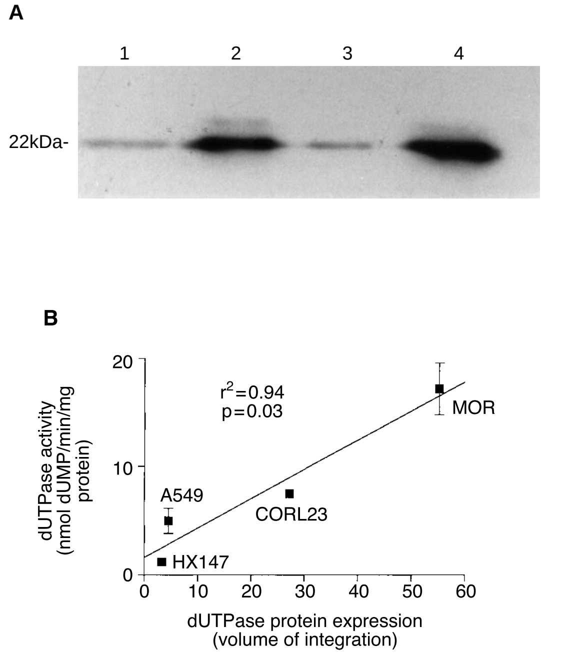

**Figure 4** (**A**) dUTPase protein expression in human lung tumour cell lines. Lane 1 A549; Lane 2 MOR; Lane 3 HX147; Lane 4 CORL23. (**B**) Correlation between dUTPase activity and dUTPase protein expression. dUTPase activity is the mean ± SD of three experiments in triplicate (except CORL23, *n* = 2). dUTPase activity in MOR cells was significantly (*P* < 0.001) higher than HX147 and A549 cells. Densitometry readings (volume of integration) are the mean of two separate observations

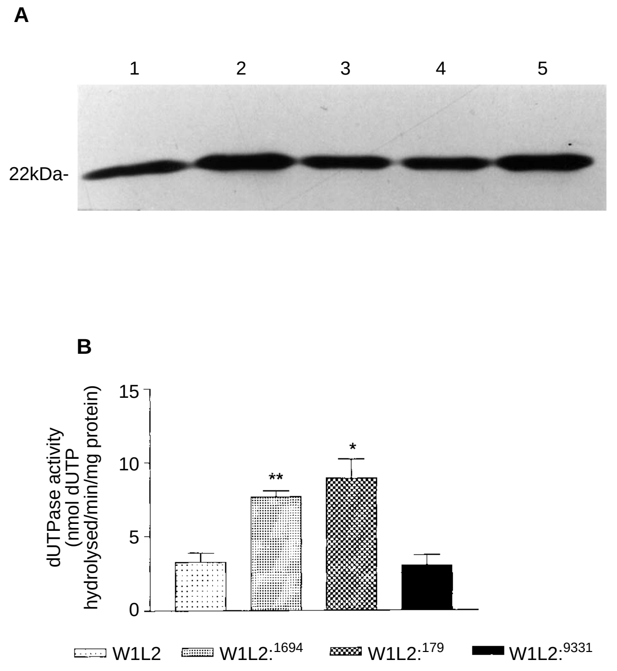

**Figure 5** (**A**) dUTPase protein expression in W1L2 resistant cell lines. Lane 1 W1L2 parent line; Lane 2 W1L2$^{1694}$; Lane 3 W1L2:Cl; Lane 4 W1L2$^{9331}$; Lane 5 W1L2$^{300179}$. (**B**) dUTPase activity in W1L2 resistant cell lines (three experiments in duplicate). **P* < 0.05; ***P* < 0.01

and possibly reflects the degree and pattern of dNTP perturbation. This in turn may be influenced by the inherent activity of key enzymes in the DNA synthetic pathway such as TS and dUTPase.

A high association ($P$ = 0.05) between TS protein and TS activity in the four lung cell lines was found. A correlation between these parameters has been reported previously (Estlin et al, 1997) but significance was only reached when two groups of cells (11 cell lines in total) were analysed together. It is generally accepted that both in vitro acquired and clinical resistance to TS inhibitors are commonly associated with overexpression of TS (Jackman et al, 1986; 1995; O'Connor et al, 1992; Johnston et al, 1994; 1995; 1997; Freemantle et al, 1995; Lenz et al, 1995; Pestalozzi et al, 1997). As may have been anticipated, the two most sensitive cell lines following a 120 h exposure (MOR and CORL23) had the lowest TS activity. However, HX147 was 5-fold more sensitive to a 120 h exposure to ZD9331 than A549 but had a higher TS activity (2-fold). Taken together, no overall correlation was observed between TS activity and sensitivity to both prolonged and short exposure to ZD9331. A lack of correlation between TS activity and sensitivity to AG337 in human tumour cell lines has been reported previously (Estlin et al, 1997). In a panel of 13 non-selected colon cancer cell lines TS activity was the best predictor of sensitivity to 5FU and 5FU/LV exposure, although for antifolates determinants of sensitivity were multifactorial (van Triest et al, 1999). In vivo studies have shown that the extent of TS inhibition affects tumour response to 5FU (Spears et al, 1984). However, depletion of dTTP pools (> 80%) (and a simultaneous increase in deoxyuridylate) following 1 μM ZD9331 showed that TS had been substantially inhibited by 24 h in all cell lines. Interestingly, in the two most sensitive lines, MOR and CORL23, dTTP had been depleted to a greater extent (~5%) than in the other two lines (10–16%).

Most of the early studies in eukaryotic cells investigating the role of dUTPase expression in cellular response to TS inhibition used mainly lymphoblastic and leukaemic cell lines (Aherne and Brown, 1999). Interpretation of these results is difficult since cells of haematopoietic origin have high dUTPase activity (Strahler et al, 1993) and do not accumulate large quantities of dUTPase following TS inhibition. dUTPase expression varied considerably in the human lung tumour cell lines studied here and similar results were observed with a panel of colorectal tumour cell lines (data not shown). dUTPase activity significantly correlated with protein expression in the lung tumour cell lines. Earlier studies have reported wide variations in dUTPase activity between tumour types (Beck et al, 1986; Strahler et al, 1993). There was a striking difference between dUTP accumulation in the lung tumour cell lines following ZD9331 treatment. A549 and HX147 cells accumulated significant quantities of dUTP whereas neither MOR nor CORL23 cells accumulated dUTP under the same conditions. The increase in the dUTP pool in A549 cells was comparable with that described using CB3717 (Curtin et al, 1991). Although no statistical correlation was found between dUTP accumulation and dUTPase activity, MOR and CORL23 had higher levels of enzyme than A549 and HX147. A non-proportional relationship between the extent of dUTP accumulation and total cellular dUTPase activity has been reported previously (Canman et al, 1993). It was suggested that this could be due to a number of factors including the non-homogeneous distribution of dUTP or dUTPase, non-linear enzyme kinetics and the accumulation of dUTP over time. Variations in deoxyuridine efflux may also exist between cell lines.

Depletion of dTTP is a lethal event but a prolonged period (at least a generation's time) is required for this to occur (Cohen, 1971; Houghton et al, 1994). If dUTP misincorporation is also an important event then a cell line with a relatively low dUTPase activity would be predicted to require a shorter duration of exposure to TS inhibitors for a lethal event to occur. An inverse relationship between intracellular dUTPase levels (which corresponded with the extent of dUTP/dTTP ratio elevation) and MTX cytotoxicity among various cell types has been reported (Beck et al, 1986). A similar trend between dUTP accumulation and sensitivity to ZD9331 was found in this study but only when the cells were exposed to drug for 24 h.

The *E. coli* dUTPase transfected HT29 human colon tumour cell line dutE7 with a ~5-fold increase in dUTPase delayed the cytotoxic effects of FdUrd (Canman et al, 1994). dutE7 is also significantly less sensitive to ZD9331-induced cytotoxicity following a 24 h exposure compared to its neotransfected control (Brown et al, 1997). This reduced sensitivity was associated with a significantly ($P < 0.05$) lower dUTP pool. However, following a 48 h exposure to ZD9331, no difference in survival between the cell lines was found. A similar observation has been reported using CB3717 and MTX (Parsels et al, 1998). Interestingly, they also reported that the expression of *E. coli* dUTPase in a HuTu80 gastrointestinal tumour cell line did not protect from FdUrd-induced DNA damage or cytotoxicity. The relatively high endogenous dUTPase activity could already be suppressing dUTP pools and it is likely that the effects of TS inhibition in HuTu80 cells, like MOR cells, is largely dUTP-independent. Lowering dUTPase activity by stable transfection of a dUTPase antisense expressing construct of the DUT-N open reading frame into HT29 cells resulted in increased dUTP accumulation, DNA damage and cytotoxicity of FdUrd (Ladner and Caradonna, 1997). The sense construct (S-4) like dutE7, was significantly less resistant to FdUrd treatment.

It is interesting that in two W1L2 cell lines with acquired resistance to TS inhibitors, an approximately 3-fold increase in dUTPase activity was observed in addition to amplified TS expression. To the best of our knowledge, this is the first time that this has been documented and suggests that dUTPase in some cell lines could be under selective pressure when exposed to TS inhibitors. This observation provides further support for the importance of dUTPase activity in mediating the effects of TS inhibition.

The results presented here show that sensitivity to ZD9331 in four human tumour cell lines may be affected by a number of factors including drug accumulation, TS activity and dTTP depletion. A clear correlation with any one factor was not obtained but this finding was probably confounded by the small number of cell lines in the panel. The observation that a large accumulation of dUTP was associated with increased sensitivity to a 24 h exposure to ZD9331 in a cell line with low dUTPase activity is consistent with previous results using dUTPase sense and antisense transfection experiments. The overexpression of dUTPase in some W1L2-resistant cell lines suggests that the activity of dUTPase may be important in protecting the cell against the effects of TS inhibition. Factors downstream of dNTP perturbation are also likely to play a role in determining response to TS inhibition in cells with different genetic backgrounds. These include the extent of DNA damage and repair, the involvement of oncogenic proteins such as bcl-2 (Fisher et al, 1993) and K-*Ras* (Houghton et al, 1998) and the ability to initiate cell death pathways (Houghton et al, 1997).

### ACKNOWLEDGEMENTS

This work was supported by the Cancer Research Campaign (Programme Grant SP2330/0201 and PhD Studentship (SD Webley))

### REFERENCES

Aherne GW and Brown SD (1999) The role of uracil misincorporation in thymineless death. In *Anticancer Drug Development Guide: Antifolate Drugs in Cancer Therapy*. AL Jackman (ed). pp 409–421. Humana Press Inc: Totowa, NJ

Aherne GW, Hardcastle A, Raynaud F and Jackman AL (1996*a*) Immunoreactive dUMP and TTP pools as an index of thymidylate synthase inhibition; effect of Tomudex (ZD1694) and a nonpolyglutamated quinazoline antifolate (CB30900) in L1210 mouse leukaemia cells. *Biochem Pharmacol* **51**: 1293–1301

Aherne GW, Hardcastle A, Ward E, Brown S and Jackman AL (1996*b*) *In vitro* and *in vivo* pharmacology of the non-polyglutamated quinazoline antifolate thymidylate synthase (TS) inhibitor, ZD9331. *Ann Oncol* **308** (7)Suppl 1: 89

Aherne GW, Hardcastle A, Brown SD, Skelton L, Findlay MPN and Jackman AL (1997) The effect of thymidylate synthase (TS) inhibitors on TS protein expression in human tumour cell lines measured by ELISA. *11th International Symposium on Chemistry and Biology of Pteridines and Folates*: 407–410

Beck WR, Wright GE, Nusbaum NJ, Chang JD and Isselbacher EM (1986) Enhancement of methotrexate cytotoxicity by uracil analogues that inhibit deoxyuridine triphosphate nucleotidohydrolase (dUTPase) activity. *Adv Exp Med Biol* **195b**: 97–104

Brown SD, Hardcastle A and Aherne GW (1997) Deoxyuridine triphosphatase (dUTPase) expression and cellular response to TS inhibitors. In *Chemistry and Biology of Pteridines and Folates*. Pfleider W and Rokos H (eds) pp 271–274. Berlin: Blackwell Science

Calvert AH, Jones TR, Dady PJ, Grzelakowska-Sztabert, Paine RM, Taylor GA and Harrap KR (1980) Quinazoline antifolates with dual biochemical loci of action. Biochemical and biological studies directed towards overcoming methotrexate resistance. *Eur J Cancer* **16**: 713–722

Canman CE, Lawrence TS, Shewach DS, Tang HY and Maybaum J (1993) Resistance to fluorodeoxyuridine induced DNA damage and cytotoxicity correlates with an elevation of deoxyuridine triphosphatase activity and failure to accumulate deoxyuridine triphosphate. *Cancer Res* **53**: 1–6

Canman CE, Radany EH, Parsels LA, Davis MA, Lawrence TS and Maybaum J (1994) Induction of resistance to fluorodeoxyuridine cytotoxicity and DNA damage in human tumour cells by expression of *Escherichia coli* deoxyuridine triphosphatase. *Cancer Res* **54**: 2296–2298

Caradonna SJ and Adamkiewicz DM (1984) Purification and properties of the deoxyuridine triphosphate nucleotidohydrolase enzyme derived from HeLa S3 cells: comparison to a distinct dUTP nucleotidohydrolase induced in herpes simplex virus-infected HeLa S3 cells. *J Biol Chem* **259**: 5459–5464

Cohen SS (1971) On the nature of thymineless death. *Ann NY Acad Sci* **106**: 292–301

Copur S, Aibak, Drake JC, Allegra CJ and Chu E (1995) Thymidylate synthase gene amplification in human colon cancer cell lines resistant to 5-fluorouracil. *Biochem Pharmacol* **49**: 1419–1426

Curtin NJ, Harris AL and Aherne GW (1991) Mechanisms of cell death following thymidylate synthase inhibition: 2 deoxyuridine 5 triphosphate accumulation, DNA damage, and growth inhibition following exposure to CB3717 and dipyridamole. *Cancer Res* **51**: 2346–2352

Estlin EJ, Balmanno K, Calvert AH, Hall AG, Lunec J, Newell DR, Pearson AD and Taylor GA (1997) The relationship between intrinsic thymidylate synthase expression and sensitivity to thymitaq in human leukaemia and colorectal carcinoma cell lines. *Br J Cancer* **76**: 1579–1585

Findlay MPN, Cunningham D, Morgan G, Clinton S, Hardcastle A and Aherne GW (1997) Lack of correlation between thymidylate synthase levels in primary colorectal tumours and subsequent response to therapy. *Br J Cancer* **75**: 903–909

 *© 2000 Cancer Research Campaign*

Fisher TC, Milner AE, Gregory CD, Jackman AL, Aherne GW, Hartley JA, Drive C and Hickman J (1993) Bcl-2 modulation of apoptosis induced by anticancer drugs-resistance to thymidylate stress is independent of classical resistance pathways. *Cancer Res* **53**: 3321–3326

Freemantle SJ, Jackman AL, Kelland LR, Calvert AH and Lunec J (1995) Molecular characterization of two cell lines selected for resistance to the folate-based thymidylate synthase inhibition, ZD1694. *Br J Cancer* **71**: 925–930

Houghton JA, Harwood FG and Houghton PS (1994) Cell cycle control processes determine cytostasis or cytotoxicity in thymineless death of colon cancer cells. *Cancer Res* **54**: 4967–4973

Houghton JA, Harwood FG and Tillman DM (1997) Thymineless death in colon carcinoma cells is mediated via Fas signaling. *Proc Natl Acad Sci USA* **94**: 8144–8149

Houghton JA, Ebanks R, Harwood FG and Tillman DM (1998) Inhibition of apoptosis after thymineless stress is conferred by oncogenic K-*Ras* in colon carcinoma cells. *Clin Cancer Res* **4**: 2841–2848

Jackman AL and Judson IR (1996) The new generation of thymidylate synthase inhibitors in clinical study. *Exp Opin Invest Drugs* **5**: 719–736

Jackman AL, Alison DL, Calvet AH and Harrap KR (1986) Increased thymidylate synthase in L1210 cells possessing acquired resistance to $N^{10}$-propargyl-5-8-dideazafolic acid (CB3717): Development, characterization, and cross resistance studies. *Cancer Res* **46**: 2810–2815

Jackman AL, Kelland LR, Kimbell R, Brown M, Gibson W, Aherne GW, Hardcastle A and Boyle FT (1995) Mechanisms of acquired resistance to the quinazoline thymidylate synthase inhibitor ZD1694 (Tomudex) in one mouse and three human cell lines. *Br J Cancer* **71**: 914–924

Jackman AL, Kimbell R, Aherne GW, Brunton L, Jansen G, Stephens TC, Smith MC, Wardleworth JM and Boyle FT (1997) Cellular pharmacology and in vivo activity of a new anticancer agent ZD9331: A water-soluble, nonpolyglutamatable, quinazoline inhibitor of thymidylate synthase. *Clin Cancer Res* **3**: 911–921

Jackson RC, Jackman AL and Calvert AH (1983) Biochemical effects of a quinazoline inhibitor of thymidylate synthetase, N-(4-(N- ((2-amino-4-hydroxy-6-quinazolinyl)-methyl)prop-2-ynylamino)benzoy)-L-glutamic acid (CB3717), on human lymphoblastoid cells. *Biochem Pharmacol* **32**: 3783–3790

Johnston PG, Fisher ER, Rockette HE, Fisher B, Wolmark N, Drake JC, Chabner BA and Allergra CJ (1994) The role of thymidylate synthase expression in prognosis and outcome of adjuvant chemotherapy in patients with rectal cancer. *J Clin Oncol* **12**: 2640–2647

Johnston PG, Lenz HJ, Leichman CG, Danenberg KD, Allegra CJ, Danenberg PV and Leichman L (1995) Thymidylate synthase gene and protein expression correlate and are associated with response to 5-fluorouracil in human colorectal and gastric tumours. *Cancer Res* **55**: 1407–1412

Johnston PG, Mick R, Recant W, Behan KA, Dolan ME, Ratain MJ, Beckman E, Weichselbaum RR, Allegra CJ and Vokes EE (1997) Thymidylate synthase expression and response to neoadjuvant chemotherapy in patients with advanced head and neck cancer. *J Natl Cancer Inst* **89**: 308–313

Ju J, Kane SE, Lenz HJ, Danenberg KD, Chu E and Danenberg PV (1998) Desensitization and sensitization of cells to fluoropyrimidines with different antisense directed against thymidylate synthase messenger RNA. *Clin Cancer Res* **4**: 2229–2236

Kobayashi H, Takemura Y, Miyachi H, Skelton L and Jackman AL (1995) Effect of hammerhead ribozyme against human thymidylate synthase on the cytotoxicity of thymidylate inhibitors. *Jpn J Cancer Res* **86**: 1014–1018

Ladner R and Caradonna S (1997) Lowering dUTPase levels through antisense induces sensitivity to fluorodeoxyuridine in HT29 cells. *Proc Am Assoc Cancer Res* **38**: 614

Ladner RD, McNulty DE, Carr SA, Roberts GD and Caradonna SJ (1996) Characterization of distinct nuclear and mitochondrial forms of human deoxyuridine triphosphate nucleotidohydrolase. *J Biol Chem* **271**: 7745–7751

Lenz HJ, Leichman CG, Danenberg KD, Danenberg PV, Grohen S, Cohen H, Laine L, Crookes P, Silberman H, Baranda J, Garcia Y, Li J and Leichman L (1995) Thymidylate synthase mRNA level in adenocarcinoma of the stomach: a predictor for primary tumour response and overall survival. *J Clin Oncol* **14**: 176–182

Lindahl T (1979) DNA glycosylases, endonucleases for apurinic/apyrimidinic sites, and base excision repair. *Prog Nucleic Acid Res Mol Biol* **22**: 135–142

O'Connor BM, Jackman AL, Crossley PH, Freemantle SE, Lunec J and Calvert AH (1992) Human lymphoblastoid cells with acquired resistance to C2-desamino-C2-methyl N10 propargyl-5, 8-dideazafolic acid: a novel folate based TS inhibitor. *Cancer Res* **52**: 1137–1143

Parsels LA, Parsels JD, Wagner LM, Loney TL, Radany EH and Maybaum J (1998) Mechanism and pharmacological specificity of dUTPase-mediated protection from DNA damage and cytotoxicity in human tumor cells. *Cancer Chemother Pharmacol* **42**: 357–362

Pestalozzi BC, Peterson HF, Gelber RD, Goldhirsch A, Fusterson BA, Trihia H, Lindtner J, Cortes-Funes H, Simmoncini E, Hyrne MJ, Golouh R, Rudenstam CM, Castiglione-Gertsch M, Allegra CJ and Johnston PG (1997) Prognostic importance of thymidylate synthase expression in early breast cancer. *J Clin Oncol* **15**: 1923–1931

Rhee MS, Wang Y, Nair MG and Galivan J (1993) Acquisition of resistance to antifolates caused by enhanced (-glutamyl hydrolase activity. *Cancer Res* **53**: 2227–2230

Skelton LA, Ormerod MG, Titley JC and Jackman AL (1998) Cell cycle effects of CB30865, a lipophilic quinazoline-based analogue of the antifolate thymidylate synthase inhibitor ICI 198583 with an undefined mechanism of action. *Cytometry* **33**: 56–66

Spears CP, Gustavsson BG, Mitchell MS, Spicer D, Berne M, Bernstein L and Danenberg PV (1984) Thymidylate synthase inhibition in malignant tumours and normal liver of patients given intraveneous 5-fluorouracil. *Cancer Res* **44**: 4144–4150

Strahler JR, Zhu X-X, Hora N, Wang YK, Andres PC, Roseman NA, Neel JV, Turka L and Hanash SM (1993) Maturation stage and proliferation-dependent expression of dUTPase in human T-cells. *Proc Natl Acad Sci USA* **90**: 4991–4995

van Triest B, Pinedo HM, van Hensbergen Y, Smid K, Telleman F, Schoenmakers PS, van der Wilt CL, van Laar JAM, Noordhuis P, Jansen G and Peters GJ (1999) Thymidylate synthase level as the main predictive parameter for sensitivity to 5-fluorouracil, but not for folate-based thymidylate synthase inhibitors, in 13 nonselected colon cancer cell lines. *Clin Cancer Res* **5**: 643–654

Webber S, Bartlett C, Boritzki TJ, Hillard JA, Howland EF, Johnston AL, Kosa M, Margosiak SA, Morse CA and Shetty DV (1996) AG337 is a novel lipophilic thymidylate synthase inhibitor: *in vitro* and *in vivo* preclinical studies. *Cancer Chemother Pharmacol* **37**: 309–317

© 2000 Cancer Research Campaign 
